# Do inhibitors of the renin–angiotensin system prevent chronic kidney disease?

**Published:** 2010-02

**Authors:** Brian Rayner

**Affiliations:** Division of Nephrology and Hypertension, University of Cape Town

The world is facing an epidemic of chronic kidney disease (CKD) and in South Africa, death rates from end-stage renal disease (ESRD) have risen by 68% from 1999 to 2006.^1^ The major drivers of this epidemic are type 2 diabetes (T2D) and/or hypertension.^2^ All major guidelines recommend ACE inhibitors (ACEIs) or angiotensin receptor blockers (ARBs) for the prevention of CKD.^3^-^5^ Recently, two important studies have reported no benefit on renal outcome in patients at high cardiovascular risk or in patients with type 1 diabetes receiving renin–angiotensin system (RAS) inhibitors,^6^,^7^ and commentators have questioned the validity of the guideline recommendations.^8^,^9^

To improve our interpretation of studies of renal disease there needs to be a better understanding of renal physiology and ‘hard’ renal versus surrogate endpoints.

## Basic renal physiology

Single nephron glomerular filtration rate (SNGFR) is determined by the following equation:

K_f_ [(*P*_gc_ – *P*_bs_) – (π_gc_ – π_bs_)]

where K_f_ is the ultrafiltration coefficient, *P*_gc_ is the glomerular capillary hydrostatic pressure, *P*_bs_ the Bowman’s space hydrostatic pressure, π_gc_ the glomerular capillary oncotic pressure and π_bs_ the Bowman’s space oncotic pressure.^10^ Total GFR is determined by the sum of all SNGFRs. Short-term changes in GFR are largely determined by changes in GC pressure, which is dependent on blood pressure (BP) and tone of the afferent and efferent renal arterioles.

In patients with CKD, there is progressive nephron loss. Overall, GFR is maintained by increasing SNGFR by raising GC pressure via afferent arteriolar dilatation and efferent vasoconstriction, largely mediated by angiotensin II. Raised GC pressure is harmful in the long term, causing progressive proteinuria and glomerulosclerosis. Therefore, BP lowering and especially lowering GC pressure by RAS inhibition has been experimentally shown to prevent glomerulosclerosis, but at the expense of short-term reduction in GFR.

In diabetes, there is evidence of GC hypertension prior to the onset of overt renal disease, and angiotensin II plays a crucial role in the pathogenesis of diabetic nephropathy.

By contrast, in patients with heart failure, there is underperfusion of the kidney, resulting in reduction of GC pressure and hence GFR. To compensate, the kidney responds by constricting the efferent arteriole and dilating the afferent arteriole to maintain GC pressure (and GFR) through tubular glomerular feedback and angiotensin II. This is termed renal autoregulation. Therefore, the use of RAS inhibitors in patients with severe cardiac failure may cause acute reduction in GFR unless there is compensatory improvement in cardiac output.

## Interpreting hard renal endpoints

In most studies, doubling in serum creatinine or ESRD is used as a hard renal endpoint. However, it is important to understand that GFR is inversely proportional to serum creatinine. Therefore, if the renal function is normal, a doubling in serum creatinine will represent a loss of 50% of renal function, i.e. 50 ml/min. However, if the GFR is 25 ml/min, a doubling in serum creatinine will also represent a loss of 50% of renal function but in absolute terms, a reduction of 12.5 ml/min. Therefore in advanced CKD, small changes in GFR may result in large increases in creatinine, giving a spurious appearance of accelerated deterioration and greater opportunity to achieve hard renal endpoints.

In most cases of CKD there is a constant, progressive loss of function of approximately 5 to 10 ml/min/year. Therefore it will take five to 10 years to demonstrate a doubling in creatinine, and 10 to 20 years for ESRD in patients with normal renal function. By contrast, in patients with reduced GFR, the time period of doubling of creatinine is considerably reduced and in the bounds of a conventional trial. Therefore trials of advanced CKD disease are usually powered to show reductions in hard renal endpoints, whereas a trial of patients with preserved renal function is not, and surrogate endpoints such as microalbuminuria and proteinuria by necessity are used.

In patients with CKD, proteinuria is an excellent surrogate marker for progression of the disease.^11^ Microalbuminuria is a predictor of incipient nephropathy in diabetics^12^ but its relationship to CKD and progression of renal disease in hypertensive patients and patients at high cardiovascular risk is less well defined and may be more of a marker of endothelial dysfunction rather than early CKD.^13^ Therefore trials reporting microalbuminuria results as an index of CKD outside diabetes must be interpreted with caution.

In patients with cardiac disease, the endpoint of doubling of serum creatinine must be seen in a different perspective, and is more an issue of safety. This may occur rapidly over hours to days due to acute reductions in renal perfusion, leading to pre-renal failure and rapidly rising creatinine, which is generally reversible if cardiac function is improved.

## Preventing kidney disease: the evidence for RAS inhibitors

## Diabetes

The natural history of diabetic nephropathy is well documented and follows a predictable course. There is usually approximately five years of hyperfiltration, 10 years of microalbuminuria with preserved renal function (or incipient nephropathy), and 10 years of overt proteinuria with declining renal function (or overt nephropathy), reaching ESRD approximately 20 to 25 years after diagnosis.^12^ Clinical trials involving diabetic patients with either normo- or microalbuminuria will require trials of 10 to 20 years’ duration to demonstrate benefits of doubling in serum creatinine and ESRD, and by definition must rely on surrogate markers of albuminuria, which fortunately in a diabetic patient is a fundamental marker for staging.^12^

Meta-analysis is generally considered the highest form of evidence. In 2004, Strippoli *et al*. published a systematic review of ACEIs and ARBs on mortality and renal outcomes in diabetic nephropathy.^14^ ARBs and ACEIs showed a highly significant 36% relative risk (RR) reduction in hard renal endpoints of doubling of serum creatinine and ESRD. In addition, on surrogate endpoints there was a 65% RR reduction in progression from microalbuminuria to macroalbuminuria, and a 3.42 greater chance of ARBs causing a regression of microalbuminuria. In an updated meta-analysis in 2008, Sarafidis *et al*. confirmed the results of the previous meta-analysis.^15^ ARBs independently confirm renal protection in type 2 diabetes, and this has been unequivocally demonstrated in the IRMA2, IDNT and RENAAL studies.^16^-^18^

However, in a recent study by Mauer *et al.*, inhibiting the RAS with either enalapril or losartan compared to placebo did not slow nephropathy but appeared to benefit retinopathy in normotensive patients with type 1 diabetes.^7^ This was a meticulously conducted study that included renal biopsies pre- and post-treatment. However, the study must be interpreted with caution. The sample size was small (about 85 patients in each group), and at baseline the mesangial fractional volume and albumin excretion rates were well within the normal ranges.^19^ Although there was significant increase in patients developing microalbuminuria in the losartan group, the mean increase in albumin excretion and mesangial fractional volume in this group was exceptionally small (4 μg/min and 0.01, respectively) and the mean values remained well within the normal range.

## Non-diabetic CKD

Similar to diabetic nephropathy, proteinuria is an excellent surrogate marker of increased risk of CKD and ESRD and may contribute directly to progression.^11^ In 2001, a meta-analysis by Jafar *et al.* demonstrated that ACEIs reduced proteinuria by 0.46 g/day and ESRD by 31%.^20^ Benefit was seen in patients with greater urinary protein at baseline, but it was doubtful if benefit extended to patients with urinary protein below 0.5 g/day. However, as it is likely that patients with less proteinuria had a slower rate of progression of renal disease, it becomes increasingly difficult to show benefits on hard renal endpoints.

In 2008, Kunz *et al.* conducted a meta-analysis of combination therapy with ACEIs and ARBs in non-diabetic CKD and showed that it further reduced proteinuria by 24 to 25% over monotherapy,^21^ but safety concerns remain. The issue is further clouded by the authenticity of the COOPERATE study,^22^ which was purported to show that combination therapy with losartan and trandolopril showed superior renal protection over individual monotherapy.^23^

## Hypertension

Although hypertension, especially systolic BP, is strongly associated with CKD,^24^ there is no evidence that BP lowering in non-diabetic hypertensives without malignant hypertension is associated with renal protection. In a meta-analysis of 10 randomised, controlled trials, treated hypertensives did not have a lower risk of renal dysfunction.^25^ On the basis of these results, should we assume that BP will have no benefit on renal outcomes? Again, if we understand that progression of CKD in non-malignant hypertension is slow, of the order of 2 to 4 ml/min/year, it would take a trial of 10 to 20 years to show benefits of antihypertensive therapy on CKD, which is far beyond the expectations of a clinical trial.

There is conflicting evidence to support the contention that ACEIs have renoprotective effects in patients with hypertension. In the ALLHAT study, there was no difference between lisinopril and chlorthalidone, which is the entirely expected result as the majority of patients had normal GFR at baseline.^26^ However, in the AASK study, ramipril appeared to be better at slowing the rate of decline in GFR compared to amlodipine in African-American patients with established hypertensive nephrosclerosis, especially those with a urinary protein/creatinine ratio above 0.22.^27^ Patients in this study had established hypertensive nephrosclerosis with a mean baseline serum creatinine that was twice normal, making renal endpoints more realisable. In a study by Wendy Hoy on Australian Aborigines, who have a very high prevalence of CKD, aggressive antihypertensive therapy, using the ACE inhibitor perindopril as a cornerstone, showed a 47% reduction in renal death.^28^

## Patients at high cardiovascular risk

The HOPE study did not show benefits of ramipril compared to placebo on hard renal endpoints in patients at high cardiovascular risk. In diabetics there was less progression of albuminuria in the ramipril arm.^29^ Of note, most patients in this study had normal renal function with a mean creatinine level of 93 μmol/l.

In the ON-TARGET study, progression of microalbuminuria was reduced by telmisartan and ramipril and combination therapy but there was a slightly greater reduction in GFR with the combination treatment.^30^ These differences in GFR over the entire clinical trial time period were very small (–6.11, –4.12 and –2.82 ml/min in the combination, telmisartan and ramipril arms, respectively, over a median of 56 months), and are in the bounds of age-related decline and therefore of questionable clinical significance. This result was also entirely predictable based on physiology. More intense inhibition of the RAS will lower GC pressure to a greater degree and reduce GFR and microalbuminuria.

Combination therapy also resulted in more cases of acute renal failure, probably due to over-treatment of BP and the effects of RAS blockade in patients with unrecognised renal artery stenosis (the estimated prevalence was about 10% in this study).

## Cardiac failure

RAS inhibitors are overwhelmingly beneficial for patients with cardiac failure with reduced ejection fraction. Renal endpoints are included for safety reasons and not to assess effects on the progression of CKD. In patients with severe cardiac failure, renal function may be critically dependent on renal auotoregulation mediated by angiotensin II, especially if patients are receiving high doses of loop diuretics. Therefore, it is anticipated that some patients receiving RAS inhibitors in cardiac failure may suffer an acute deterioration in renal function and have a doubling in serum creatinine due to acute pre-renal failure, which is generally reversible with restoration of cardiac function.

It is often a delicate juggling act to prevent pulmonary congestion and maintain renal perfusion with optimal doses of loop diuretics, aldosterone antagonists, beta-blockers and ACEIs. Renal function must be carefully monitored as well as serum potassium (K^+^) levels. Therefore, for Onuigbo, in his critical re-appraisal of the evidence for RAS blockade on renal outcomes, to use cardiac failure studies to conclude that these agents may not be renoprotective is extremely difficult to understand.^8^

## Do RAS inhibitors improve cardiovascular outcomes in patients with CKD and proteinuria?

Balamuthusamy *et al.* conducted a meta-analysis of cardiovascular outcomes in patients with CKD and proteinuria receiving RAS blockers. They showed a significant reduction in risk of myocardial infarction, heart failure and total cardiovascular outcomes compared with placebo, and reduced cardiovascular outcomes and heart failure when compared to control treatment.^31^

## Are there safety concerns about RAS inhibitors in patients with CKD and cardiovascular disease?

Not unlike any pharmaceutical agents, RAS inhibitors need to be used with due diligence. RAS inhibitors are contraindicated in pregnancy and may cause hyperkalaemia, especially in patients with severe cardiac disease or advanced CKD, particularly if combined with aldosterone anatagonists. Careful monitoring of the serum K^+^ is required.

It is also well established that RAS inhibitors may cause an acute deterioration in renal function. Patients at risk are those with severe cardiac failure, bilateral renal artery stenosis, normotensive patients with CKD, and patients with CKD or cardiac failure who become pre-renal due to overuse of diuretics or inter-current illnesses such as acute gastroenteritis. On the other hand, from a physiological perspective, RAS inhibitors cause a rise in creatinine due to a reduction in GC pressure. One of the most frequently asked questions of nephrologists is ‘What degree of rise in creatinine level is acceptable after introducing an RAS inhibitor?’

Bakris and Weir found that in most trials there was minimal or no rise in creatinine levels with initiation of ACEIs in patients with normal renal function, but if there was pre-existing renal insufficiency (creatinine ≥ 124 μmol/l), up to a 30% decline in creatinine levels may be acceptable and the ACE inhibitor continued, provided there was no further deterioration and the serum K^+^ concentration was not above 5.6 mmol/l.^32^ In fact, this finding predicted a long-term preservation of renal function. In my opinion, any rise in creatinine above 20% should prompt the physician to consider renal artery stenosis or pre-renal insufficiency. Because of these safety concerns, RAS inhibitors should preferably be initiated and monitored by specialists in patients with abnormal renal function, especially if estimated GFR is below 45 ml/min, whereas the general practitioner can safely handle patients with normal renal function.

## Conclusion

There is unequivocal evidence that RAS inhibitors protect the kidney in patients with diabetes, in non-diabetics with CKD, particularly if urinary protein is above 0.5 g/day, and improve cardiovascular outcomes in patients with proteinuric CKD. There is suggestive evidence that ACEIs may preserve renal function in patients with hypertension, especially if there is established hypertensive nephrosclerosis. The role of RAS inhibitors in the general protection of the kidney in patients at high cardiovascular risk remains unproven, but this must be interpreted in the context that these trials are not powered to make any positive or negative conclusions. RAS inhibitors may cause acute deterioration in renal function in patients with bilateral renal artery stenosis and severe cardiac failure. Initiation of RAS inhibition in any patient who has CKD with elevated creatinine must be carefully monitored.
